# Physiological Correlates of the Yo-Yo Intermittent Recovery Tests in Canadian Male Professional Soccer Players

**DOI:** 10.3390/sports14090384

**Published:** 2026-09-01

**Authors:** Riccardo Bucciarelli, Farzad Yousefian, Ethan Brown, Lawrence L. Spriet, Margaret Jones, John Srbely

**Affiliations:** 1 Department of Human Health and Nutritional Sciences (HHNS), College of Biological Sciences, University of Guelph, Guelph, ON N1G 2W1, Canada; lspriet@uoguelph.ca (L.L.S.); jsrbely@uoguelph.ca (J.S.); 2 Research Center in Sports Sciences and Human Development (CIDESD), Department of Sports Sciences, University of Beira Interior, 6201-001 Covilha, Portugal; farzad.yousefian@ubi.pt; 3 FPF Academy, Portuguese Football Federation, 1495-433 Oeiras, Portugal; 4 Faculty of Kinesiology, Sport, and Recreation (KSR), College of Health Sciences, University of Alberta, Edmonton, AB T6G 2H9, Canada; embrown@ualberta.ca; 5 College of Education and Human Development, George Mason University, Fairfax, VA 22030, USA; mjones15@gmu.edu

**Keywords:** association football, high-intensity running, global positioning system, hierarchical regression, performance analysis, sports recruitment

## Abstract

This cross-sectional observational study examined associations between distance covered in the Yo-Yo Intermittent Recovery Test Level 1 (YYIRTL1) and Level 2 (YYIRTL2) and selected physical and physiological performance characteristics in Canadian male professional soccer players. A convenience sample of 29 players from one Canadian Premier League club completed YYIRTL1, YYIRTL2, VO_2_max testing, the Cunningham–Faulkner Test (CFT), squat-jump-derived power, theoretical running-power index, maximal running velocity (Vmax), reactive strength index (RSI), and isometric mid-thigh pull (IMTP). Pearson correlations with 95% confidence intervals and Benjamini–Hochberg false-discovery-rate adjustment were calculated. Hypothesis-driven hierarchical regression models were limited to VO_2_max and CFT. YYIRTL1 distance was positively correlated with VO_2_max (r = 0.666) and CFT (r = 0.540), and was inversely correlated with squat-jump-derived power and theoretical running-power index (both r = −0.501). VO_2_max explained 44.3% of YYIRTL1 variance; adding CFT increased explained variance to 52.9%. YYIRTL2 distance was positively correlated with CFT (r = 0.740) and VO_2_max (r = 0.684). CFT explained 54.8% of YYIRTL2 variance; adding VO_2_max increased explained variance to 72.2%. These findings suggest that VO_2_max and CFT performance are associated with YYIRT performance in this single-club professional cohort. Because the sample was small and externally unvalidated, the regression models should be interpreted as exploratory explanatory models rather than prospective prediction tools.

## 1. Introduction

The Yo-Yo Intermittent Recovery Test, Level 1 (YYIRTL1) and Level 2 (YYIRTL2) are commonly used to assess the fitness of athletes, including soccer players [1]. Performance on the YYIRTL1 has previously been correlated with aerobic fitness [2,3], anaerobic fitness [4,5], running velocity [6], and agility [7] in athletes, including soccer players, and it has also been shown to be highly correlated with the amount of high-intensity running performed in soccer match play [8,9]. The YYIRTL2 has also been shown to have similar correlations to the distance covered at high intensities during a soccer match and similar reliability as the YYIRTL1 [2], as well as greater sensitivity to training than the YYIRTL1 [10]. The YYIRTL2 can also be completed in less time than the YYIRTL1, which may make it preferable to many athletes [11]. Performance on the test has also previously been correlated with aerobic fitness [12,13,14], and repeated sprint ability [14,15], and the test has also been shown to utilize the anaerobic energy system to a much greater degree than the YYIRTL1, with lower muscle glycogen, lower muscle pH, and higher blood lactate (LA) concentration post-test, as well as greater rates of both muscle and blood LA accumulation, than the YYIRTL1 [2]. Some research has also demonstrated small or moderate correlations between the YYIRTL2 and other measures of anaerobic fitness, including maximal running velocity (Vmax) and agility (Reactive Strength Index/RSI) [14], while other research has shown no association [16,17].

While both YYIRT tests provide measures of the interplay between several different components of physical and physiological fitness, the test scores alone do not provide insight into the relative contribution of each fitness component to overall test performance. Although many studies have demonstrated associations between one or more components of fitness and YYIRT tests in soccer players, no research currently exists that examines the correlations between the tests and a broader list of outcome measures addressing all physiological abilities relevant for soccer players. Similarly, the predictive ability of a comprehensive set of aerobic, anaerobic, and phosphagenic fitness outcomes to YYIRT performance has not been addressed.

The purpose of the present study was to investigate the relationship between various components of fitness, and performance on the YYIRT tests. The primary hypotheses were that VO_2_max would show the strongest association with YYIRTL1 distance and that CFT performance would show the strongest association with YYIRTL2 distance. Secondarily, exploratory hypotheses were that Vmax, squat-jump-derived power, theoretical running-power index, RSI, and IMTP would be associated with both YYIRT outcomes. A further objective was to examine whether a limited, hypothesis-driven hierarchical regression approach using VO_2_max and CFT could explain variance in YYIRTL1 and YYIRTL2 distance.

Improved knowledge of the roles of physiological qualities in YYIRT performance would benefit clinicians and trainers as it provides them with important insight into developing targeted training programmes for professional soccer players. Clarifying these associations may help practitioners interpret YYIRT performance with greater specificity. However, because the study was cross-sectional, involved a small convenience sample from only one professional club, and did not include external validation, the analyses are intended to be explanatory and hypothesis-generating rather than definitive prediction models for recruitment or player selection.

## 2. Materials and Methods

### 2.1. Study Design, Setting, and Ethics

This cross-sectional observational study was conducted during the 2022 Canadian Premier League pre-season. The study was approved by the University of Guelph Research Ethics Board (REB 19-05-044) and conducted in accordance with the Declaration of Helsinki. All participants provided written informed consent before testing.

### 2.2. Participants

A convenience sample of 29 male professional-level soccer players (age 23.2 ± 2.5; height 179 ± 7.2 cm; weight 76.5 ± 7.63 kg) from the Halifax Wanderers Football Club was recruited between 28 December 2021 and 15 January 2022. Inclusion criteria were being under contract for the 2022 season and being physically healthy and able to participate in fitness testing and team training. Exclusion criteria were not under contract with the club or having a current injury or other limitations preventing participation in high-intensity testing. All eligible athletes were approached, no eligible athletes declined participation, and all recruited athletes completed every assessment. No a priori recruitment calculation was performed because recruitment involved the available professional team cohort only. Participants completed an informed consent form and a Physical Activity Readiness Questionnaire Plus (PAR-Q+) before testing [18].

### 2.3. Testing Procedures and Timeline

Testing was completed over four consecutive testing days, at the beginning of the pre-season training period, from 31 January to 3 February 2022. The fixed testing sequence was: Day 1, squat jump, drop jump, IMTP, and CFT; Day 2, YYIRTL1; Day 3, VO_2_max; Day 4, 1080 sprint load–velocity testing, Vmax, and YYIRTL2. All tests were conducted as part of the club’s pre-season testing schedule. Participants also underwent low-to-moderate intensity team training following completion of testing on Days 1 and 2, and no other team training was conducted on Days 3 and 4. The fixed order was retained for logistical reasons and is acknowledged as a limitation because residual fatigue, familiarization, and unequal preceding loads may have influenced some outcomes, particularly the YYIRTL2 on Day 4.

Squat jump and squat-jump-derived power. Vertical ground-reaction force was recorded at 1000 Hz using a Hawkin Dynamics force platform (Hawkin Dynamics, Westbrook, ME, USA). The force plates were factory calibrated by the manufacturer and were zeroed without external load prior to testing each athlete. Force-platform outcomes were obtained using the manufacturer’s software; no additional user-applied filtering of the force-time signal was performed. Following zeroing, participants then performed three squat-jump trials, separated by 60 s of recovery. Each athlete maintained a static flexed-knee position for approximately 2 s before jumping vertically as high as possible without countermovement or arm swing [19]. Athletes were not given any instruction about jumping technique, as previous research has indicated that subjects chose the depth that maximized both peak force and peak velocity resulting in maximal power output [20]. The best trial (highest jump) was retained for analysis. Jump height was estimated from flight time assuming equal take-off and landing height: jump height (m) = g × flight time^2^/8. Squat-jump-derived power was estimated using the Sayers equation based on jump height and body mass [21]: estimated jump power (W) = 60.7 × jump height (cm) + 45.3 × body mass (kg) − 2055. Because force-time data were not available for reanalysis, the Sayers-derived value was retained as an estimated mechanical power measure rather than a direct measure of anaerobic energy-system capacity.

Drop jump and reactive strength index (RSI). Once again, force plates were zeroed without external load prior to testing each athlete. Each participant performed three drop jumps from a 42 cm box onto the force plate with hands kept out of the movement and instructions to minimize ground-contact time while jumping as high as possible. 60 s of rest was provided between each jump trial. The reactive Strength Index (RSI) was calculated as jump height divided by ground-contact time [22]. The best valid trial, with the highest RSI value, was used for analysis.

Isometric mid-thigh pull. Force plates were once again zeroed without external load prior to testing each athlete. Participants stood on the force plate with a 20.4 kg Olympic barbell pinned in a squat rack at approximately mid-thigh height. Three maximal IMTP trials were performed with 60 s of rest between trials. Participants were instructed to pull as hard as possible for approximately 2 s following a verbal countdown [23]. The trial producing the greatest maximal voluntary force was used for analysis. The source dataset recorded IMTP relative to body mass (N·kg^−1^). Because several details required for full replication, including precise joint angles, pre-tension criteria, and force-onset procedures, were not available retrospectively, this limitation was acknowledged.

Cunningham–Faulkner Test (CFT). Participants completed the Cunningham–Faulkner anaerobic test on Day 1. The treadmill was set at 12.9 km·h^−1^ and 20% incline. Timing began when the athlete started running unsupported and ended when the athlete could no longer maintain the required speed. Time to exhaustion was recorded to the nearest 0.5 s by the same trained researcher [24]. A 15 min cool-down followed the test.

YYIRTL1 and YYIRTL2. YYIRTL1 was completed on Day 2 and YYIRTL2 was completed on Day 4 on an indoor turf soccer field. Each test consisted of repeated 2 × 20 m shuttle runs performed to audio cues, with 10 s active recovery periods between shuttles. Distance covered was recorded according to the standardized YYIRT protocol [25].

VO_2_max testing. On Day 3, athletes completed graded exercise testing using a portable cardiometabolic system (PNOĒ, Athens, Greece), which has been validated against the COSMED Quark CPET, a previously validated stationary cardiometabolic testing system [26]. Prior to each athlete’s test, the PNOĒ system underwent the manufacturer’s standard software-guided calibration procedure. Following warm-up, athletes completed a graded exercise test comprising 5 running stages, with each stage comprising a 2 min interval of running at progressively increasing running speeds, beginning at 12.9 kph and increasing by 1.6 kph per stage, until a speed of 19.3 kph in the fifth and final stage. Graded treadmill-based exercise tests using similar 2 min running stages, with a total test duration of 8–12 min, have previously been shown to elicit reliable VO_2_max values [27]. Breath-by-breath gas-exchange data were processed using the manufacturer’s software. The highest processed oxygen uptake value was retained as VO_2_max when a plateau or secondary maximal-effort criteria were met; otherwise, the value was interpreted cautiously as the highest observed oxygen uptake. Maximal heart rate, respiratory-exchange ratio, perceived exertion, and full stage-completion data were not available for all players retrospectively and are therefore acknowledged as limitations.

Load–velocity profile, theoretical running-power index, and Vmax. On Day 4, participants performed three 30 m linear sprint trials using the 1080 Sprint (1080 Motion, Lidingö, Sweden) on indoor synthetic turf, with 2 min of rest between trials. Prior to beginning testing on Day 4, the 1080 Sprint was calibrated once according to the manufacturer’s recommended procedure, including calibration of the tether position to establish the measurement reference point. External loads of 1, 8, and 15 kg were used to derive a load–velocity profile, and the theoretical running-power index was calculated from the fitted load–velocity relationship [28]. Theoretical maximal running power was determined as the product of load x velocity, with the selection of the load which decreased maximal velocity by 50%; this decrease in velocity, 50%Vdec, has been previously shown to elicit maximal power during resisted sprinting and load-velocity profiling [29]. Because the source dataset recorded this variable in arbitrary units, it is interpreted as a theoretical running-power index rather than a directly measured mechanical power output. Maximal 5 m running velocity was retained as Vmax. Following completion of resisted sprint trials, all athletes were provided with 30 min of recovery, prior to performing the YYIRTL2.

### 2.4. Statistical Analysis

Distributional characteristics were evaluated using visual inspection and formal tests as appropriate. Pearson product–moment correlations were calculated between each YYIRT outcome and VO_2_max, CFT performance, squat-jump-derived power, theoretical running-power index, Vmax, RSI, and IMTP. Correlation coefficients are reported with 95% confidence intervals. Because multiple correlations were examined for each YYIRT test, Benjamini–Hochberg false-discovery-rate (FDR) adjusted *p* values were also calculated. Statistical significance was set at α = 0.05; correlations other than the hypothesis-driven VO_2_max and CFT analyses were interpreted as exploratory.

A hypothesis-driven hierarchical linear regression approach was utilized. Models were limited to VO_2_max and CFT, the two variables identified a priori as the expected primary correlates of YYIRTL1 and YYIRTL2, respectively. For YYIRTL1, VO_2_max was entered in Model 1 and CFT was added in Model 2. For YYIRTL2, CFT was entered in Model 1 and VO_2_max was added in Model 2. Unstandardized coefficients (B), standard errors (SE), standardized coefficients (β), t statistics, *p* values, and 95% confidence intervals were calculated. Model fit was summarized using R^2^ and adjusted R^2^, and the incremental contribution of the second variable was evaluated using ΔR^2^ and the F-change test.

Regression assumptions were evaluated using residual diagnostics, including residual normality, homoscedasticity, linearity/model specification, multicollinearity, leverage, and influential observations. Variance inflation factor (VIF) and tolerance were used to assess multicollinearity. Internal model performance was evaluated using leave-one-out cross-validation (LOOCV), with cross-validated R^2^, root mean square error (RMSE), and mean absolute error (MAE) reported. Repeated shuffled 5-fold cross-validation (1000 repetitions) was used as an additional robustness analysis. Given the finite sample of professional players available from one club, the multivariable analyses were considered exploratory.

## 3. Results

Data from all 29 players were included in the analyses, with complete data available for all variables used in the revised models (Table 1). Mean YYIRTL1 distance was 2710.3 ± 460.3 m and mean YYIRTL2 distance was 1229.0 ± 363.3 m. Mean VO_2_max was 61.1 ± 3.2 mL·kg^−1^·min^−1^ and mean CFT performance was 64.8 ± 9.9 s. IMTP was recorded in the source dataset as force relative to body mass (41.4 ± 5.3 N·kg^−1^), rather than absolute force in newtons.

### 3.1. Correlational Analyses

**Figure 1:**
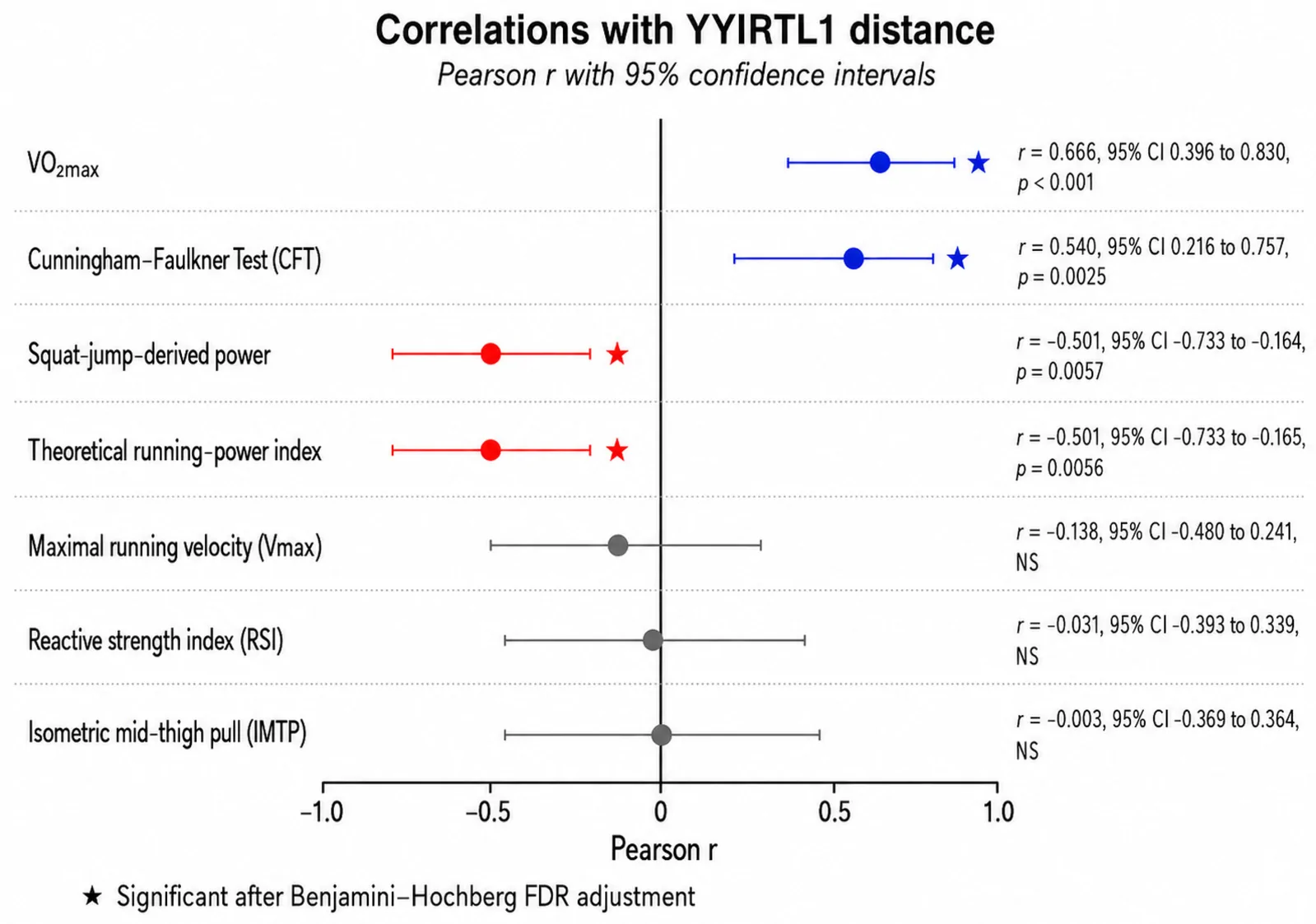
Correlations between YYIRTL1 distance and selected physiological and physical-performance characteristics in male professional soccer players. VO_2_max and Cunningham–Faulkner Test (CFT) performance were positively associated with YYIRTL1 distance, whereas squat-jump-derived power and the theoretical running-power index were negatively associated; these four relationships remained significant after Benjamini–Hochberg false-discovery-rate adjustment. Blue markers and error bars indicate significant positive correlations, red markers and error bars indicate significant negative correlations, and grey markers and error bars indicate non-significant correlations. Error bars represent 95% confidence intervals, and the star indicates significance after FDR adjustment.

For YYIRTL1, VO_2_max showed a positive correlation with distance covered (r = 0.666, 95% CI 0.396 to 0.830, *p* < 0.001), as did CFT performance (r = 0.540, 95% CI 0.216 to 0.757, *p* = 0.0025). Squat-jump-derived power (r = −0.501, 95% CI −0.733 to −0.164, *p* = 0.0057) and the theoretical running-power index (r = −0.501, 95% CI −0.733 to −0.165, *p* = 0.0056) were inversely correlated with YYIRTL1 distance. Vmax, RSI, and IMTP were not significantly correlated with YYIRTL1. The four associations with VO_2_max, CFT, squat-jump-derived power, and running-power index remained significant after Benjamini–Hochberg FDR adjustment (Figure 1).

**Figure 2:**
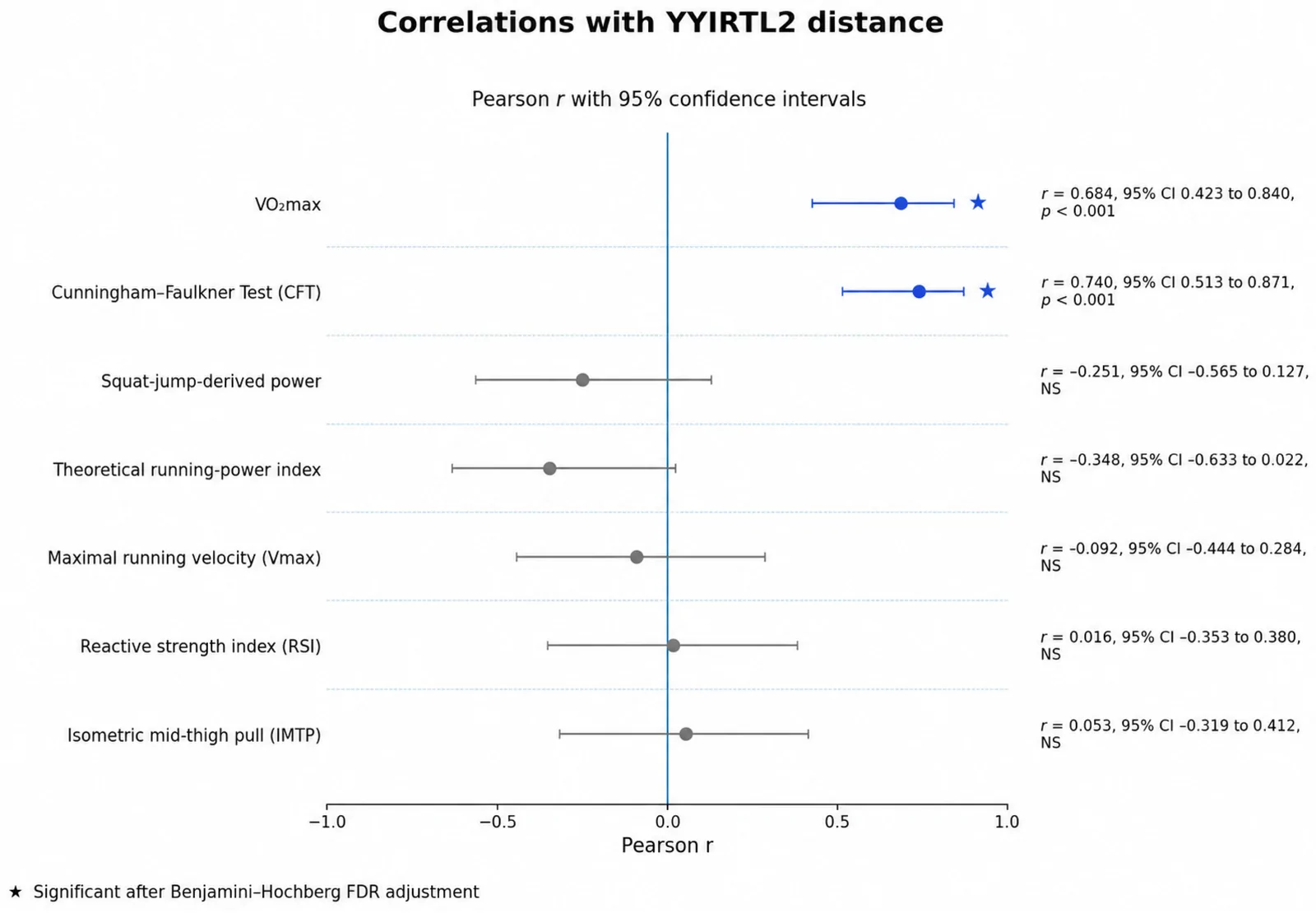
Correlations between YYIRTL2 distance and selected physiological and physical-performance characteristics in male professional soccer players. VO_2_max and Cunningham–Faulkner Test (CFT) performance were positively associated with YYIRTL2 distance and remained significant after Benjamini–Hochberg false-discovery-rate adjustment, whereas squat-jump-derived power, theoretical running-power index, maximal running velocity (Vmax), reactive strength index (RSI), and isometric mid-thigh pull (IMTP) were not significantly correlated with YYIRTL2 performance. Blue markers and error bars indicate significant positive correlations, whereas grey markers and error bars indicate non-significant correlations. Error bars represent 95% confidence intervals, and the star indicates significance after FDR adjustment.

For YYIRTL2, CFT performance showed the strongest positive correlation with distance covered (r = 0.740, 95% CI 0.513 to 0.871, *p* < 0.001), followed by VO_2_max (r = 0.684, 95% CI 0.423 to 0.840, *p* < 0.001). The correlations with squat-jump-derived power, theoretical running-power index, Vmax, RSI, and IMTP were not statistically significant. CFT and VO_2_max remained significant after Benjamini–Hochberg FDR adjustment (Figure 2).

### 3.2. Hierarchical Regression

In Model 1 for the YYIRTL1, VO_2_max explained 44.3% of the variance in YYIRTL1 distance (R^2^ = 0.443, adjusted R^2^ = 0.423; F (1, 27) = 21.49, *p* < 0.001). VO_2_max was positively associated with YYIRTL1 distance (B = 97.06, SE = 20.94, β = 0.666, t = 4.64, *p* < 0.001, 95% CI 54.10 to 140.03). Adding CFT performance in Model 2 increased explained variance to 52.9% (R^2^ = 0.529, adjusted R^2^ = 0.493; F (2, 26) = 14.61, *p* < 0.001). The increase in explained variance was ΔR^2^ = 0.086 (F-change (1, 26) = 4.75, *p* = 0.0385). In the final model, VO_2_max (B = 77.90, SE = 21.50, β = 0.534, t = 3.62, *p* = 0.0012, 95% CI 33.71 to 122.10) and CFT (B = 14.88, SE = 6.82, β = 0.321, t = 2.18, *p* = 0.0385, 95% CI 0.85 to 28.91) were both associated with YYIRTL1 distance (Table 2).

Final explanatory equation for YYIRTL1 distance was:

YYIRTL1 distance (m) = −3014.89 + 77.90 (VO_2_max) + 14.88 (CFT).

In Model 1 for the YYIRTL2, CFT performance explained 54.8% of the variance in YYIRTL2 distance (R^2^ = 0.548, adjusted R^2^ = 0.531; F (1, 27) = 32.76, *p* < 0.001). CFT performance was positively associated with YYIRTL2 distance (B = 27.05, SE = 4.73, β = 0.740, t = 5.72, *p* < 0.001, 95% CI 17.35 to 36.75). Adding VO_2_max in Model 2 increased explained variance to 72.2% (R^2^ = 0.722, adjusted R^2^ = 0.701; F (2, 26) = 33.82, *p* < 0.001). The increase in explained variance was ΔR^2^ = 0.174 (F-change (1, 26) = 16.31, *p* < 0.001). In the final model, CFT (B = 20.22, SE = 4.14, β = 0.553, t = 4.89, *p* < 0.001, 95% CI 11.72 to 28.72) and VO_2_max (B = 52.63, SE = 13.03, β = 0.457, t = 4.04, *p* < 0.001, 95% CI 25.84 to 79.42) were both associated with YYIRTL2 distance (Table 3).

Final explanatory equation for YYIRTL2 distance was:

YYIRTL2 distance (m) = −3297.88 + 20.22 (CFT) + 52.63 (VO_2_max).

### 3.3. Model Diagnostics and Internal Validation

For the final YYIRTL1 model, residual normality was not rejected (Shapiro–Wilk *p* = 0.110), and the Breusch-Pagan test yielded no evidence of heteroscedasticity (*p* = 0.681). No model misspecification was found from the Ramsey RESET test (*p* = 0.124). Similarly, for the final YYIRTL2 model, residual normality was also not rejected (Shapiro–Wilk *p* = 0.814), and there was also no evidence of heteroscedasticity (Breusch–Pagan *p* = 0.938) or model misspecification (Ramsey RESET *p* = 0.478). VO_2_max and CFT showed limited collinearity (r = 0.409); VIF was 1.20 and tolerance was 0.833 for both variables. Influence diagnostics identified individual observations warranting inspection, but LOOCV refitting did not change the direction of either coefficient.

LOOCV yielded a cross-validated R^2^ of 0.430 for YYIRTL1 (RMSE = 341.6 m; MAE = 262.3 m) and 0.670 for YYIRTL2 (RMSE = 205.2 m; MAE = 159.7 m). Shuffled 5-fold cross-validation was performed across 1000 repetitions and mean cross-validated R^2^ values were 0.415 for YYIRTL1 (RMSE = 345.6 m; MAE = 264.2 m) and 0.663 for YYIRTL2 (RMSE = 207.0 m; MAE = 162.3 m). The new, internally validated values were lower than the apparent R^2^ values and are presented in order to more accurately interpret the strength of the model.

### 3.4. Sensitivity Analysis for Body-Mass Scaling

The source dataset showed that IMTP had already been normalized to body mass (N·kg^−1^). As a sensitivity analysis, squat-jump-derived power was also expressed relative to body mass. Relative squat-jump power remained inversely correlated with YYIRTL1 (r = −0.523, 95% CI −0.746 to −0.193, *p* = 0.0036) and YYIRTL2 (r = −0.438, 95% CI −0.693 to −0.086, *p* = 0.017). The theoretical running-power index variable was not additionally normalized because the measure is recorded in arbitrary units.

## 4. Discussion

### 4.1. Principal Findings

The findings of the current study supported our primary hypotheses that VO_2_max would have the strongest association with YYIRTL1 performance, and that the Cunningham-Faulkner Test would have the strongest association with YYIRTL2 performance. The main finding was that YYIRTL1 and YYIRTL2 distances were most consistently associated with VO_2_max and CFT performance in this cohort of professional male soccer players. The exploratory hypotheses concerning Vmax, RSI, and IMTP were not supported. Squat-jump-derived power and the theoretical running-power index were inversely associated with YYIRTL1, but these associations should be interpreted cautiously because they may reflect sample-specific characteristics rather than direct physiological mechanisms.

### 4.2. Comparison with Previous Research

Professional male soccer players are required to perform between 150 and 250 high-intensity repetitions every game, with limited recovery time between each repetition [30]. The average length of sprints in male professional soccer is 8–12 m, and the average time duration is 1–2 s [31], and most of these intense movements occur at crucial moments, such as scoring opportunities, which may have a significant impact upon the outcome of the game [32]. High levels of anaerobic phosphagenic capacity, including speed, strength, and agility, are necessary for optimal execution of repeated high-intensity tasks while high aerobic and anaerobic capacities are also required for optimal recovery between activities. The positive association between VO_2_max and YYIRTL1 is consistent with evidence that intermittent running tests depend partly on aerobic capacity and the ability to recover between repeated high-intensity efforts [2,4]. Although the links between aerobic capacity and YYIRTL1 performance are clear, research examining the correlations between anaerobic fitness and YYIRTL1 performance in male players is equivocal. For example, Rampinini et al. [33] found strong correlations between blood LA, hydrogen ions (H^+^), bicarbonate (HCO_3_^−^) and the rate of LA accumulation (all assessed during performance of a treadmill-based high-intensity running test) with YYIRTL1 performance in male adult professional and amateur soccer players. Similarly, Chuman et al. [4] found correlations between YYIRTL1 performance and the anaerobic Wingate test in elite male youth Under-13 soccer players, but not in elite Under-17 male professional academy players. In the current study, both aerobic and anaerobic fitness were correlated with YYIRTL1 performance.

Previous literature has also demonstrated very strong correlations between the YYIRTL2 and anaerobic fitness [2,10,15], and strong correlations with aerobic fitness [2,3,12,14] in male professional soccer players. The positive association between CFT performance and both YYIRT outcomes is also consistent with the high-intensity nature of these tests, particularly YYIRTL2, which begins at a higher speed and may impose greater anaerobic and local muscular demands. The present study also adds to and reinforces the previous literature demonstrating links between both anaerobic and aerobic fitness and the YYIRTL2, as it is the first to include hierarchical regression analysis, used to determine and quantify the ability of these measures of fitness to predict performance on the YYIRTL2.

The present findings do, however, contradict some previous research emphasizing the importance of certain anaerobic phosphagenic qualities, including running velocity and agility outcome measures, to performance on the YYIRTL1 and YYIRTL2 in soccer players. Ingebrigtsen et al. [15] reported moderate correlations between average running velocity (Vavg) and YYIRTL1 performance in male professional soccer players. Sanchez-Garcia et al. [34] similarly reported a moderate correlation (r = 0.40) by investigating the relationship between Vmax (assessed using global positioning satellite or GPS) and high-intensity running performance on the YYIRTL1. In contrast, other studies have reported no correlations between YYIRTL1 and Vavg in soccer players [16,35]; however, one of these studies [16] used female rather than male soccer players, while both used collegiate rather than professional soccer players. An additional study [36] reported no correlation between Vavg over a 15-metre sprint and YYIRTL1 in male professional players. Previous research has also shown no association between the YYIRTL2 and Vmax or agility in female collegiate soccer players [16] and male professional youth soccer players [17]; however, more recent work with male professional soccer players did find small and moderate correlations between the YYIRTL2 and acceleration, Vmax, and agility [14]. The YYIRT tests do involve high-speed running with changes of direction within each shuttle run, which suggests that running velocity and agility may be important physical qualities for optimal performance of the tests, but thus far, results both from our research and other research remain inconclusive, and further research is needed to resolve this question.

Research investigating the correlations between YYIRT tests and other anaerobic phosphagenic fitness qualities has primarily focused on power, with several studies reporting a significant positive correlation between maximal power (Pmax) and YYIRTL1 performance in soccer players [3] and other athletes [5,6,37,38]. However, in these studies Pmax was determined using non-running-derived measures such as a Wingate cycle ergometer [5], a force plate during squat jump and/or countermovement jump tests [3,6,37], or both [37]. The extrapolation of these findings may be limited to running performance in soccer players, and both Kilit and Arslan [37] (tennis) and Banda et al. [38] (basketball) did not assess soccer players in their research. Moderate correlations have also been reported between indirect jump-derived measures of power, including squat jump and countermovement jump, and YYIRTL2 performance (r = 0.29 and r = 0.33, respectively) [14]. Based on these findings, tests with greater specificity to running, and tests done with soccer players as opposed to other team sport athletes, will provide more insight into the relationships between anaerobic phosphagenic fitness qualities and YYIRT performance in soccer players.

### 4.3. Interpretation of Negative Associations with Power Variables

In the present study, inverse associations between YYIRTL1 performance and squat-jump-derived power, and the theoretical running-power index were found. This finding was unexpected, as previous research reported strong relationships between power and the YYIRTL1 [3,5,6,36,37], and the YYIRTL2 in athletes [14]. The findings, however, should not be interpreted as evidence that greater power directly impairs YYIRT performance. Alternative explanations include body-mass scaling, positional composition, measurement error, multicollinearity among neuromuscular variables, fatigue from the fixed testing order (especially on Day 4), and small-sample instability. The sensitivity analysis using body-mass-normalized squat-jump power showed a similar direction of association, but this does not eliminate the possibility that the result is sample specific. Muscle-fibre distribution or training specialization may be plausible contributors, but these variables were not measured directly and should therefore be considered speculative.

### 4.4. Strengths, Limitations, Future Research, and Practical Applications

A strength of this study was the recruitment of professional players and the assessment of YYIRTL1 and YYIRTL2 alongside laboratory and field-based measures of aerobic, high-intensity treadmill, sprint, jump, reactive-strength, and isometric-strength performance. Studying both YYIRT levels in the same cohort provides applied information for practitioners who use these tests within professional soccer environments.

The current study is not without limitations. Firstly, the fitness of male professional soccer players in the current study was assessed from only one team, which includes a relatively small sample of players (only 3 or 4) in each playing position. The links between aerobic fitness measured in a lab setting and repeated high-intensity game sequences in elite Italian male professional soccer players have been shown to vary greatly by player position [39], and activity profiles in elite English male professional soccer players, including the distances covered at high intensities during match play, have been shown to vary considerably among different playing positions [40]. Collectively, these findings highlight the need for further research assessing the relationships between various components of fitness and the YYIRT tests in larger samples, thereby allowing for multiple players in each position to be assessed.

Second, although the exploratory hierarchical regression models predicted 53% of the variance in YYIRTL1 performance, and 72% of the variance in YYIRTL2 performance, 47% of the YYIRTL1, and 28% of the YYIRTL2, respectively, remain unaccounted for. Repeated sprint ability (RSA), the ability to perform repeated, maximal intensity efforts, with brief recovery periods, over an extended period [41] was not tested in the current study. While RSA has been previously correlated to VO_2_max [33], research examining the correlations between RSA and the YYIRTL1 remains inconclusive. Ingebrigtsen et al. [15] did find moderate to large correlations, depending upon the sprint distance used in the RSA test, with the strongest correlations found between 35-metre RSA and YYIRTL1, and only moderate correlations found between 10- and 20-metre RSA and YYIRTL1. The authors speculated that the stronger correlations observed with longer sprint distances were reflective of the greater contribution from the aerobic energy system required to perform and recover from sprints at these distances.

Regarding the contribution of RSA to the YYIRTL2, a previous study included a measure of RSA (6 × 2 × 20 m shuttle-run sprints, with 20 s of recovery between sprints) in the assessment protocol and found a strong correlation (r = 0.59) between RSA and YYIRTL2 performance [14]. Interestingly, in their analysis, the authors first assessed the ability of the linear and change of direction speed tests (5 and 20 m sprint, and 5-0-5 agility test) to predict the RSA score, and then, the ability of the RSA to predict YYIRTL2 performance. The authors concluded that linear running speed and change-of-direction speed are the main predictors of RSA, and that RSA is one of the two main predictors of the YYIRTL2 [14]. Given the fact that RSA has been shown to be a unique physical quality, differentiated from the aerobic and anaerobic qualities tested for in the present study, and that RSA has been shown to be related to performance in both YYIRT tests, further research using a similar model and sample of Canadian male professional soccer players, and including a test of RSA, may allow for a greater percentage of the variance in YYIRT tests to be accounted for.

Other important limitations of the present study include the low events-per-variable ratio, the absence of external validation for regression analyses, incomplete retrospective reporting for selected testing procedures, possible selection bias, and fatigue or order effects from the fixed testing sequence. Future research should examine larger, multi-club samples, include positional stratification, externally validate explanatory models, and incorporate repeated-sprint ability and match-running outcomes. From a practical perspective, VO_2_max and CFT performance may help practitioners interpret YYIRT performance, but the present results should not be used as stand-alone recruitment or talent-identification criteria because YYIRT performance reflects only one component of overall soccer performance.

## 5. Conclusions

Findings of this cross-sectional, single-club cohort of male professional soccer players, indicated that VO_2_max and CFT performance were associated with YYIRTL1 and YYIRTL2 distance. VO_2_max and CFT together explained variance in both YYIRT outcomes, with stronger explanatory performance for YYIRTL2 than YYIRTL1. In contrast, Vmax, RSI, and IMTP were not significantly associated with either YYIRT outcome, and negative associations involving power variables should be interpreted cautiously as exploratory and potentially sample-specific. These findings support further externally validated research on physiological and physical-performance correlates of YYIRT performance, but the present models should not be applied as stand-alone clinical, coaching, recruitment, or talent-identification tools.

## Figures and Tables

**Table 1:** Descriptive statistics for participant characteristics and physiological and physical-performance variables. Values are mean (SD) and 95% confidence intervals for the mean. CFT = Cunningham–Faulkner Test; IMTP = isometric mid-thigh pull; RSI = reactive strength index; Vmax = maximal running velocity; a.u. = arbitrary units.

	Age (yrs)	Height (cm)	Body Mass (kg)	YYIRTL1 Distance (m)	YYIRTL2 Distance (m)	VO_2_max (mL·kg^−1^·min^−1^)	CFT (s)	IMTP (N·kg^−1^)	RSI	Squat-Jump Power (W)	Theoretical Running-Power Index (a.u.)	Vmax (m·s^−1^)
Mean (SD)	23.2 (2.5)	179.9 (7.2)	76.51 (7.63)	2710.3 (460.3)	1229.0 (363.3)	61.12 (3.16)	64.79 (9.94)	41.37 (5.30)	2.57 (0.59)	4458.4 (522.3)	105.1 (11.4)	8.66 (0.46)
95% CI	22.2 to 24.2	177.2 to 182.6	73.61 to 79.42	2535.3 to 2885.4	1090.8 to 1367.2	59.92 to 62.32	61.01 to 68.58	39.35 to 43.38	2.34 to 2.79	4259.7 to 4657.1	100.8 to 109.4	8.49 to 8.84

**Table 2:** Hierarchical multiple regression predicting YYIRTL1 distance.

Model	Predictor	B	SE	β	T	*p*	95% CI for B	R^2^	Adjusted R^2^	ΔR^2^
1	VO_2_max	97.06	20.94	0.666	4.64	<0.001	54.10 to 140.03	0.443	0.423	—
2	VO_2_max	77.90	21.50	0.534	3.62	0.0012	33.71 to 122.10	0.529	0.493	0.086
	Cunningham–Faulkner Test (CFT)	14.88	6.82	0.321	2.18	0.0385	0.85 to 28.91			

Model statistics. Model 1: F (1, 27) = 21.49, *p* < 0.001. Model 2: F (2, 26) = 14.61, *p* < 0.001. Addition of CFT in Model 2 produced ΔR^2^ = 0.086, F-change (1, 26) = 4.75, *p* = 0.0385. *Note.* B = unstandardized regression coefficient; SE = standard error; β = standardized regression coefficient; CI = confidence interval; ΔR^2^ = change in explained variance.

**Table 3:** Hierarchical multiple regression predicting YYIRTL2 distance.

Model	Predictor	B	SE	β	T	*p*	95% CI for B	R^2^	Adjusted R^2^	ΔR^2^
1	Cunningham–Faulkner Test (CFT)	27.05	4.73	0.740	5.72	<0.001	17.35 to 36.75	0.548	0.531	—
2	Cunningham–Faulkner Test (CFT)	20.22	4.14	0.553	4.89	<0.001	11.72 to 28.72	0.722	0.701	0.174
	VO_2_max	52.63	13.03	0.457	4.04	<0.001	25.84 to 79.42			

Model statistics. Model 1: F (1, 27) = 32.76, *p* < 0.001. Model 2: F (2, 26) = 33.82, *p* < 0.001. Addition of VO_2_max in Model 2 produced ΔR^2^ = 0.174, F-change (1, 26) = 16.31, *p* < 0.001. *Note.* B = unstandardized regression coefficient; SE = standard error; β = standardized regression coefficient; CI = confidence interval; ΔR^2^ = change in explained variance.

## Data Availability

The data presented in this study are available from the corresponding author on reasonable request, subject to confidentiality restrictions related to professional team and player data. Public deposition was considered but was not possible without additional consent for open sharing of individual athlete data.

## Abbreviations

YYIRTL1	Yo-Yo Intermittent Recovery Test Level 1
YYIRTL2	Yo-Yo Intermittent Recovery Test Level 2
CFT	Cunningham–Faulkner Test
FDR	False discovery rate
IMTP	Isometric mid-thigh pull
LOOCV	Leave-one-out cross-validation
MAE	Mean absolute error
RMSE	Root mean square error
RSI	Reactive strength index
VIF	Variance inflation factor
Vmax	Maximal running velocity
LA	Lactic Acid
pH	Power of Hydrogen
REB	Research Ethics Board
PARQ	Physical Activity Readiness Questionnaire
cm	Centimetres
kg	Kilograms
